# Super-resolution generative adversarial networks with static T2*WI-based subject-specific learning to improve spatial difference sensitivity in fMRI activation

**DOI:** 10.1038/s41598-022-14421-5

**Published:** 2022-06-20

**Authors:** Junko Ota, Kensuke Umehara, Jeff Kershaw, Riwa Kishimoto, Yoshiyuki Hirano, Yasuhiko Tachibana, Hisateru Ohba, Takayuki Obata

**Affiliations:** 1 Medical Informatics Section, Department of Medical Technology, QST Hospital, National Institutes for Quantum Science and Technology (QST), 4-9-1 Anagawa, Inage-ku, Chiba, 263-8555 Japan; 2 Applied MRI Research, Department of Molecular Imaging and Theranostics, Institute for Quantum Medical Science, National Institutes for Quantum Science and Technology, Chiba, Japan; 3 Quantum-Medicine AI Research Group, QST Advanced Study Laboratory, National Institutes for Quantum Science and Technology, Chiba, Japan; 4 Research Center for Child Mental Development, Chiba University, Chiba, Japan

**Keywords:** Somatosensory system, Software

## Abstract

The spatial resolution of fMRI is relatively poor and improvements are needed to indicate more specific locations for functional activities. Here, we propose a novel scheme, called Static T2*WI-based Subject-Specific Super Resolution fMRI (STSS-SRfMRI), to enhance the functional resolution, or ability to discriminate spatially adjacent but functionally different responses, of fMRI. The scheme is based on super-resolution generative adversarial networks (SRGAN) that utilize a T2*-weighted image (T2*WI) dataset as a training reference. The efficacy of the scheme was evaluated through comparison with the activation maps obtained from the raw unpreprocessed functional data (raw fMRI). MRI images were acquired from 30 healthy volunteers using a 3 Tesla scanner. The modified SRGAN reconstructs a high-resolution image series from the original low-resolution fMRI data. For quantitative comparison, several metrics were calculated for both the STSS-SRfMRI and the raw fMRI activation maps. The ability to distinguish between two different finger-tapping tasks was significantly higher [p = 0.00466] for the reconstructed STSS-SRfMRI images than for the raw fMRI images. The results indicate that the functional resolution of the STSS-SRfMRI scheme is superior, which suggests that the scheme is a potential solution to realizing higher functional resolution in fMRI images obtained using 3T MRI.

## Introduction

Functional magnetic resonance imaging (fMRI) has rapidly developed into an essential tool for neuroscientific research. Functional neuroimaging tools visualize the regions of the brain responsible for specific cognitive functions^1^. In comparison to other cognitive imaging modalities, such as electroencephalography (EEG), electrocorticography (ECoG), near-infrared spectroscopy (NIRS), magnetoencephalography (MEG), and positron emission tomography (PET)^1^, fMRI is the most extensively used. One advantage of fMRI is that it has a relatively high spatial resolution^2^. However, the spatial resolution of general anatomical MRI images is higher (pixel size < 1 mm × 1 mm) than that of general fMRI data (pixel size < 3 mm × 3 mm). It is therefore desirable to improve the spatial resolution of fMRI so that more specific locations for functional activities can be identified^3^. Although 7 Tesla (7T) MRI scanners can acquire MRI images of higher resolution than those acquired by 3T MRI, they are also more expensive and have limited availability^4^. Therefore, a method that can be used to obtain higher resolution maps of brain responses from 3T fMRI data is desirable^4,5^.

A possible solution to this problem is to apply a deep learning-based super-resolution technique^6^ to translate the low-resolution images acquired with a 3T MRI scanner into high-resolution images^7^. Deep learning-based super-resolution (SR) schemes have shown high performance both qualitatively and quantitatively when applied to medical imaging^8^. Recently, these schemes have been improved by combining the SR technique with generative adversarial networks (GANs)^9^ to form SRGANs^10^. A SRGAN facilitates the generation of more realistic images than simple convolutional neural network-based (CNN-based) SR techniques^11–13^. To generate a high spatial resolution fMRI series from low-resolution data, a source of high spatial resolution information is required. Static T2*-weighted images (T2*WI) and gradient-echo EPI fMRI data exhibit similar contrast because fMRI relies on T2* relaxation^14^. Since T2*WI can be acquired at high spatial resolution, we focus here on static T2*WI as the images needed to train an SRGAN for fMRI.

In this study we have developed a new GAN-based SR scheme for fMRI, called Static T2*WI-based Subject-Specific Super Resolution fMRI (STSS-SRfMRI), to enhance the functional resolution of fMRI. The key element of the proposed method is the utilization of static T2*-WI obtained from each subject in order to train a subject-specific model. This study aims to assess the enhancement of functional resolution using the STSS-SRfMRI scheme in comparison to the results obtained from the raw unprocessed fMRI images (raw fMRI).

## Materials and methods

### Subjects

Adhering to the Declaration of Helsinki, informed consent was obtained in writing from all participants prior to participation. The experimental protocols, which were approved by the Institutional Review Board at the National Institutes for Quantum and Radiological Science and Technology, conformed to the safety guidelines for MRI research.

A total of 35 healthy female volunteers (mean age 26.9 ± 6.7 years) with no history of neurological disease were selected as candidates for this study. The data from five subjects were excluded for the following reasons: the image data were damaged due to a technical error (1 subject), the candidate was visually impaired and unable to perform the task appropriately (1 subject), there were severe motion artifacts (1 subject), and the candidate failed to perform the task satisfactorily for indeterminate reasons (2 subjects).

### MRI data acquisition

All subjects underwent a 3T MRI scan with a MAGNETOM Verio scanner (Siemens AG; Munich, Germany). fMRI scanning was performed using a gradient-echo echo-planar imaging (GE-EPI) sequence (echo time: 25 ms, repetition time: 500 ms, flip angle: 44°, field-of-view: 1440 mm × 1440 mm, acquisition matrix: 64 × 64, slice thickness: 4 mm, slices: 30, total scans: 900) during a finger-tapping task. In addition, T2*WI were acquired using a two-dimensional (2D) rapid gradient-echo sequence (echo time: 25 ms, repetition time: 2000 ms, flip angle: 90°, field-of-view: 240 mm × 240 mm, acquisition matrix: 128 × 128 and 64 × 64, slice thickness: 4 mm, number of slices: 30). Furthermore, T1-weighted MRI images were acquired using a three-dimensional (3D) magnetization-prepared rapid gradient-echo sequence (echo time: 1.98 ms, repetition time: 2300 ms, flip angle: 9°, field-of-view: 250 mm × 250 mm, acquisition matrix: 256 × 256, slice thickness: 1 mm). Table 1 shows the parameters of the fMRI, T2*-weighted MRI, and T1-weighted MRI scans.

**Table 1:** Magnetic resonance imaging scan parameters.

	Matrix	Total volumes	Repetition time [ms]	Echo time [ms]	Flip angle [degrees]
Functional magnetic resonance imaging	64 × 64	900	500	25	44
Low spatial resolution T2*weighted imaging	64 × 64	1	2000	25	90
High spatial resolution T2*weighted imaging	128 × 128	1	2000	25	90
T1 weighted imaging	256 × 256	1	2300	1.98	9

### Finger-tapping procedure

A finger-tapping task was performed during fMRI scanning. Supplementary Figure 1 outlines the task protocol, which included phases of tapping either the thumb or little finger of one hand and resting phases between each task. Prior to beginning the experiment, participants were given sufficient time to familiarize themselves with the tasks and select which hand they would use for tapping. The instructions on which finger to tap or rest were provided on a screen behind the participant’s head, and were viewed through a mirror mounted on the head coil. The projection was presented using E-prime 1.0 (Psychology Software Tools, PA, USA). Each subject was instructed to tap the cued finger, but not the adjacent fingers, at their own pace.

### Functional analysis

Before functional analysis, the first 60 scans were excluded from the analysis to ensure that the magnetization reached equilibrium^15^. After coregistration of the T1WI structured data to the automated anatomical labeling (AAL) atlas^16^, the functional data was coregistered to the T1WI data. The transformations were then combined to identify the motor area in the functional data sets. In addition, linear trends in the time series were removed, and the noise level was reduced by applying a low-pass filter to each pixel. Spatial filtering was also applied using a Gaussian filter with $\sigma =1.5$.

After this preprocessing, functional activation maps were obtained from the image time series by correlating the signal intensity time-course of each pixel with an on/off task design convolved with a canonical hemodynamic response function. SPM12 (revision 7219)^17^ was used for the analysis. The cross-correlation (CC) coefficient was calculated for each pixel using

1 $$CC=\frac{\overrightarrow{{R}_{x}}\cdot \overrightarrow{{R}_{y}}}{\left|\overrightarrow{{R}_{x}}\right|\left|\overrightarrow{{R}_{y}}\right|},$$

where $\overrightarrow{{R}_{x}}$ is the reference task design and $\overrightarrow{{R}_{y}}$ is the signal intensity time-course of the pixel^15^. All image preprocessing and functional analysis was performed in MATLAB R2018b (Mathworks, Natick, MA, USA).

#### Deep learning-based super-resolution

**Figure 1:**
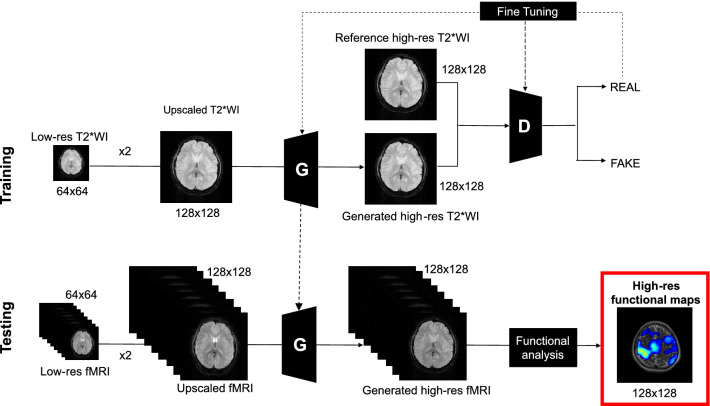
Overview of the Static T2*WI-based Subject-Specific Super Resolution fMRI (STSS-SRfMRI) scheme proposed in this study. The upper and lower parts correspond to the training and testing phases, respectively. In the training phase, the generator (G) was optimized to form a relationship between the low-resolution and high-resolution T2*WI. The discriminator (D) made a decision whether the input was “real” (i.e., the reference high-res T2*WI) or “fake” (i.e., the generated high-res T2*WI). G learned to generate more realistic output via feedback from D. In the testing phase, a high-resolution functional MRI (fMRI) time series was reconstructed from the low-resolution fMRI data using the optimized generator, and subsequently a high-resolution functional map was calculated based on the high-resolution fMRI.

Figure 1 depicts an overview of the proposed method. The STSS-SRfMRI scheme includes two unique ideas: first, it uses high spatial resolution static T2*WI as the training data; second, it applies subject-specific learning. As described in the introduction, the static T2*WI were used to introduce high spatial resolution information into the training process. Also, as functional signal changes are usually quite small, subject-specific learning was used to eliminate any anatomical variation that might be artificially introduced by including T2*WI data from other subjects.

Before training, the pixel intensity of the T2*WI training data was adjusted and scaled to match the intensity of the fMRI data. All 30 slices of the T2*WI data from each subject were used for training and validation to build a subject-specific model. The trained model was then applied to the fMRI data from the same subject.

The SRGAN used in this work was customized in several ways. Rather than using an up-sampling block in the generator G, the low resolution images were upscaled to a 128 × 128 matrix size using lanczos 3 interpolation^18,19^ before being input. All the batch normalization layers were also removed^20^. A discriminator (D) was applied with the number of convolutional layers set to 10 to accommodate the size of the input. We implemented the modified SRGAN network using an adaptive moment estimation (Adam) optimizer with an initial decay rate of 0.9, a scaling factor of 2, patch size of 64, batch size of 2, an initial learning rate of 0.0001, and 100,000 iterations. The training images were the 30 slices of the corresponding T2*WI data. The experiments were implemented in PyTorch 1.1.0 on Ubuntu 16.04 LTS.

### Identifying the neural activation-related region

**Figure 2:**
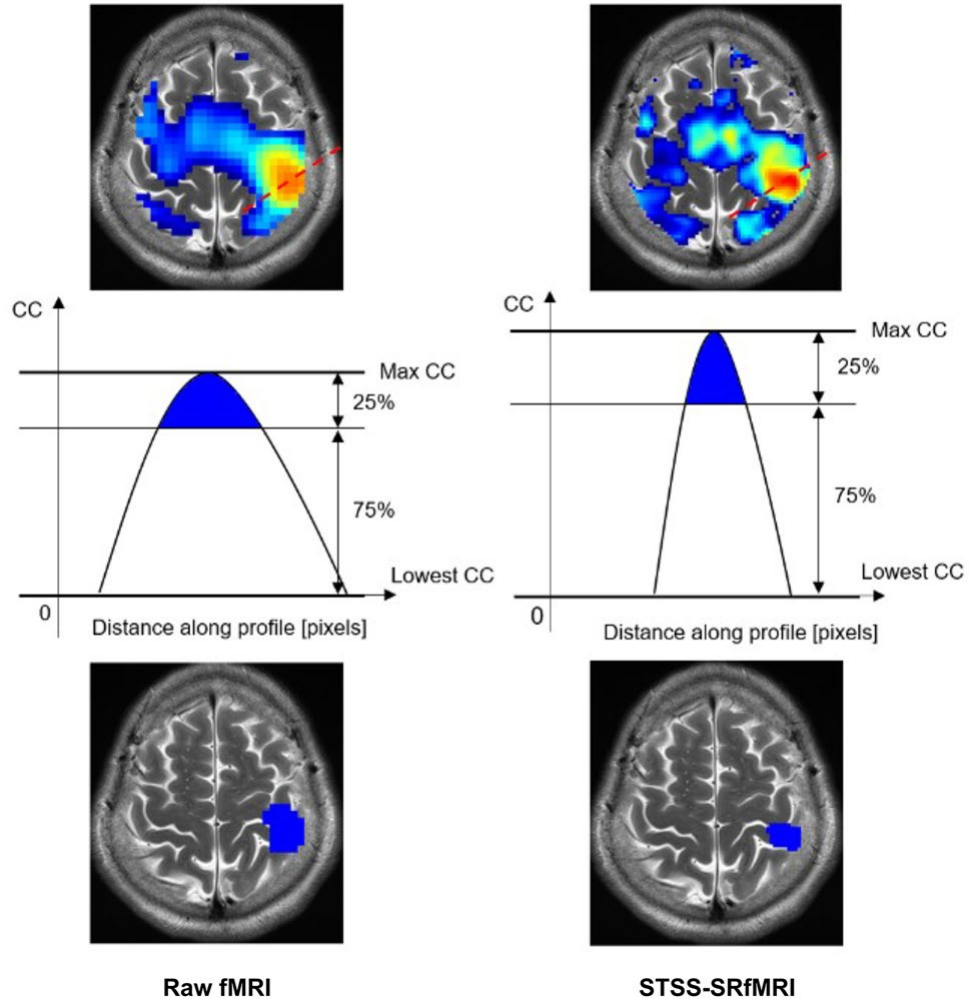
Overview of how the activation-related region was defined for each tapping task. First the activation maps were obtained from the raw and the Static T2*WI-based Subject-Specific Super Resolution fMRI (STSS-SRfMRI) image series (top row). Second, the top 25% between the max and minimum CC values was set as the threshold (middle row). Finally, the region consisting of pixels having values equal to or higher than the threshold value was defined as the activation-related region (bottom).

The activation maps generated from the low-resolution fMRI data (the raw map) and from the processed output of the STSS-SRfMRI scheme (STSS-SR fMRI map), were compared based on how effectively they localized the activation region. For this purpose, the regions corresponding to the thumb and little finger activation tasks were separately identified for the raw fMRI and STSS-SRfMRI maps of each subject. First, a CC map was calculated for each input image series (i.e., the raw or STSS-SR data) for each subject and each activated finger. Second, the activation-related region in each CC map was defined as the region consisting of pixels having values equal to or above a threshold value, see Fig. 2. The threshold value was defined as

2 $$Threshold=maxCC-\frac{maxCC-minCC}{4}.$$

The number of pixels included in the activation-related region of the raw fMRI map was compared to that of the STSS-SR fMRI map for each finger of each subject. As the STSS-SR fMRI maps had pixels that were four times smaller than those of the raw fMRI maps for the same sized area, the number of pixels in the STSS-SR fMRI maps was divided by 4 before comparison.

### Independence of the extracted activated regions for the different tasks

**Figure 3:**
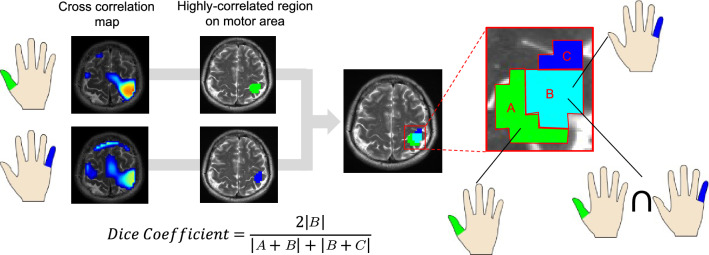
Definition of the Dice coefficient used in this study to assess how clearly the activated regions corresponding to the thumb and the little finger tasks were separated. The Dice coefficient was calculated for the extracted activation-related regions of the thumb (green) and little finger (blue) for each subject. The light-blue area corresponds to the overlap between the activation-related regions for the thumb and little finger.

The raw fMRI and STSS-SR fMRI maps obtained in the previous sub section were compared to determine which of them has a higher functional resolution for the thumb and little finger tasks. For this purpose, a Dice coefficient^21,22^ was calculated for the extracted activation-related regions of the thumb and little finger for each subject (Fig. 3). This assessment was based on the well-known fact that the motor function areas for the thumb and little finger are not the same^23,24^.

### Statistical analysis

The number of pixels included in each activation-related region, and the Dice coefficient calculated from the raw fMRI and STSS-SR fMRI maps were statistically compared using the Wilcoxon signed-rank test (p < 0.05 was considered significant). The EZR graphical interface to R version 3.5.2^25^, was used to make these statistical comparisons.

## Results

### Identifying the neural activation-related region

**Figure 4:**
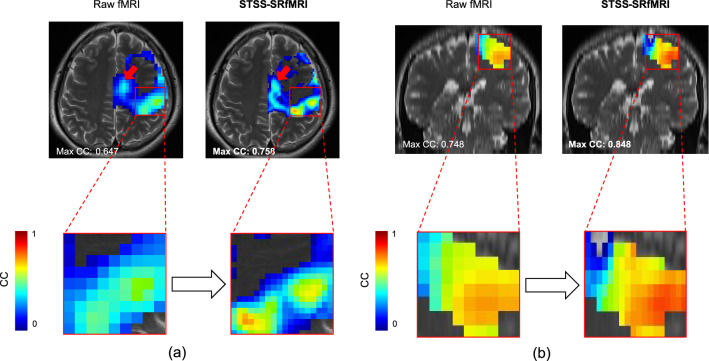
Example comparing the cross-correlation (CC) maps obtained from the raw unpreproccessed fMRI data and the Static T2*WI-based Subject-Specific Super Resolution fMRI (STSS-SRfMRI) for one subject: (**a**) the maps in the raw axial plane, and (**b**) the sagittal plane reconstruction for the same case. The areas having the highest CC values in the primary motor cortex are magnified below to showcase the details. In a visual comparison, the highly correlated area in the STSS-SRfMRI maps occupied a relatively limited area in comparison to the raw fMRI map. The red arrows in (**a**) point to the supplementary motor cortex, which appears more sharply defined in the STSS-SRfMRI maps.

**Figure 5:**
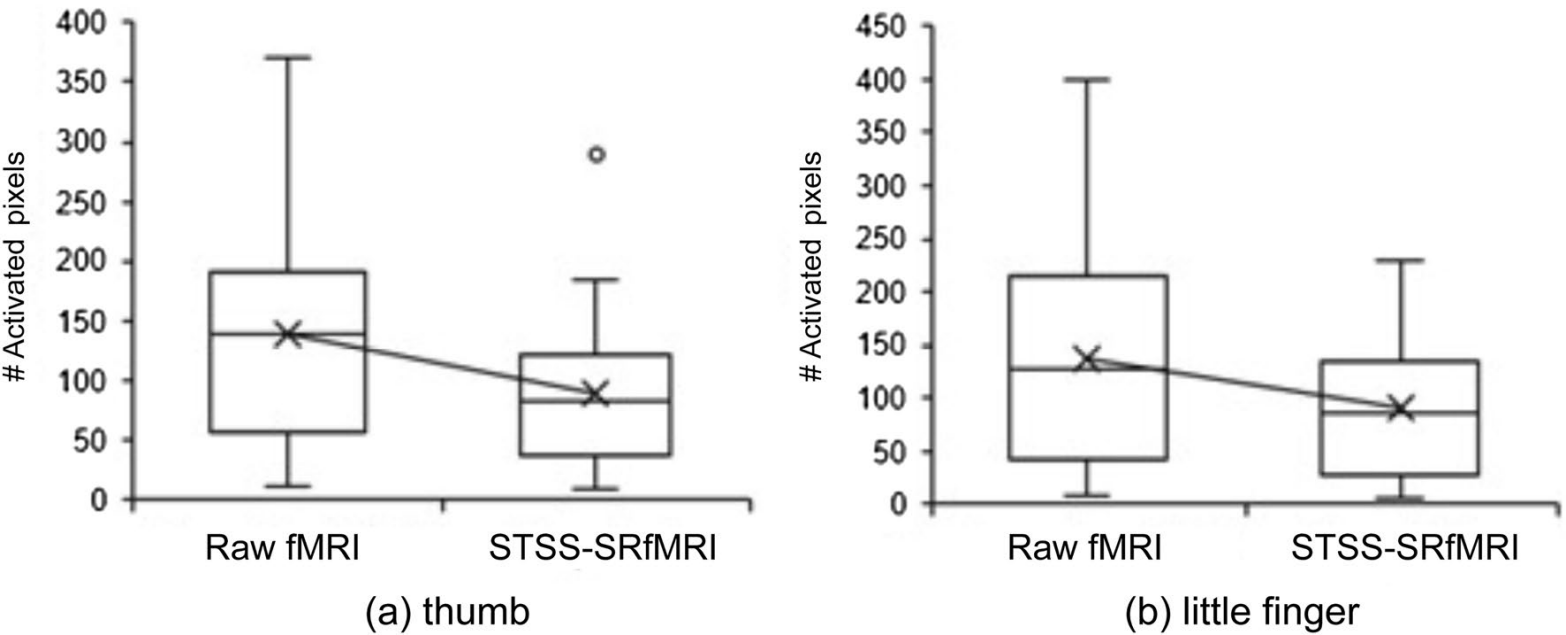
Comparison of the number of activated pixels extracted from the raw fMRI maps and the Static T2*WI-based Subject-Specific Super Resolution fMRI (STSS-SRfMRI) for the (**a**) thumb and (**b**) little finger. The STSS-SRfMRI maps yielded significantly fewer pixels than the raw fMRI maps for both the thumb (p < 0.001) and little finger (p < 0.001). The median (interquartile range (IQR)) for the raw fMRI and STSS-SRfMRI maps were 140.00 (64.75–184.00) and 83.37 (42.31–119.68), respectively, for the thumb and 128.50 (43.50–208.25) and 87.00 (28.93–129.00), respectively, for the little finger.

Figure 4 presents representative examples of the CC maps obtained via analysis of the raw unpreprocessed and STSS-SRfMRI processed data. The STSS-SRfMRI method appears to enhance the functional resolution. Figure 5 compares the number of pixels in the activation-related regions of the motor areas corresponding to thumb-tapping and little finger-tapping. The activation-related regions extracted from the STSS-SRfMRI maps had significantly fewer pixels than those extracted from the raw fMRI maps for both the thumb (p < 0.001) and little finger (p < 0.001) tasks.

### Independence of the extracted activated regions for the different tasks

**Figure 6:**
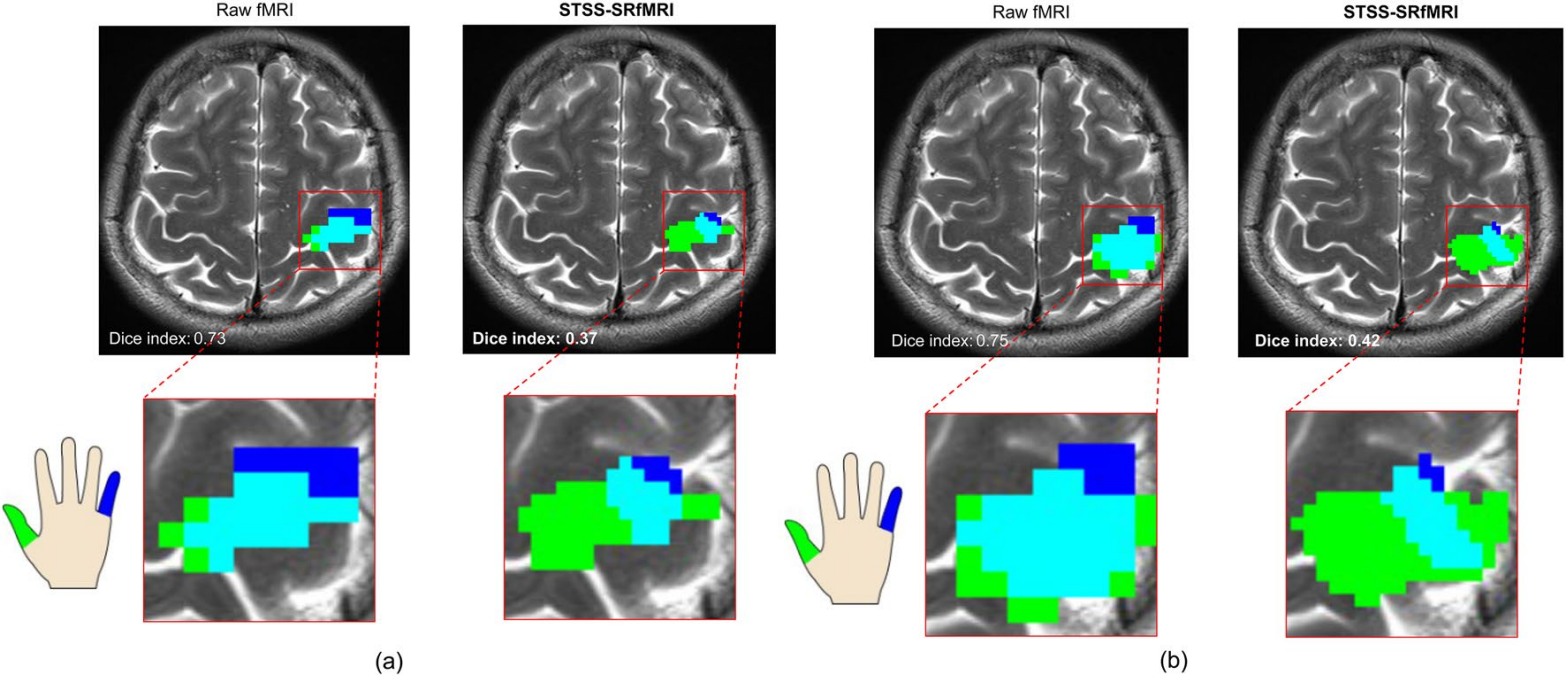
Two examples of the distribution of the activation-related regions. The colored voxels indicate regions that are highly correlated with only thumb-tapping (green), with only little finger-tapping (blue), and with both thumb and little finger tapping (light-blue). On visual inspection, the light-blue regions corresponding to the Static T2*WI-based Subject-Specific Super Resolution fMRI (STSS-SRfMRI) scheme are narrower than those of the raw fMRI maps, suggesting improved functional resolution.

**Figure 7:**
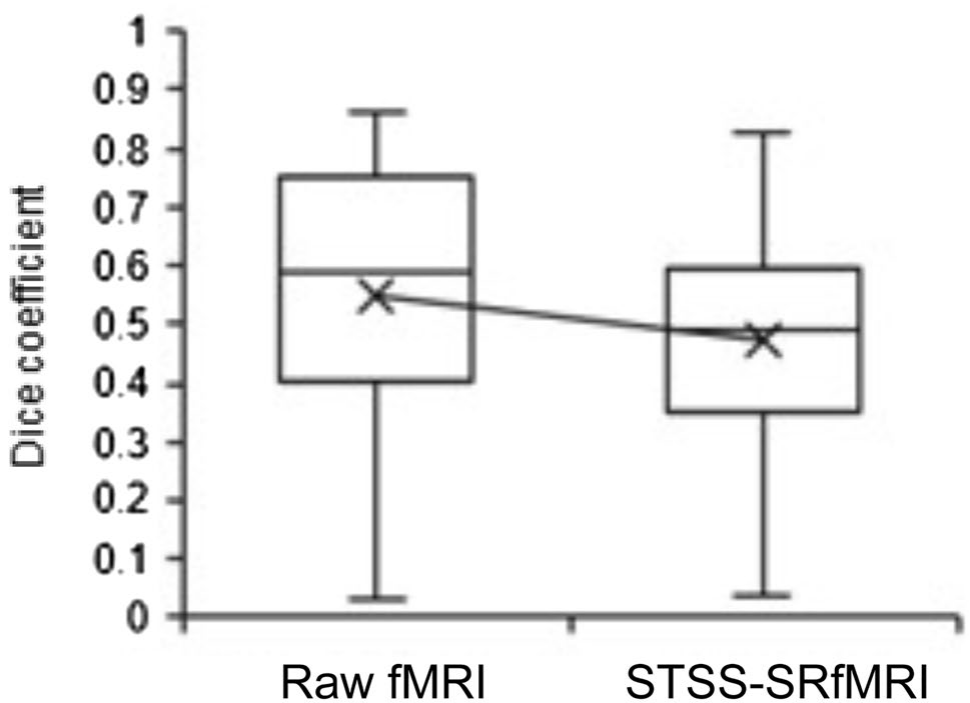
Comparison of the Dice coefficients for the raw fMRI and the Static T2*WI-based Subject-Specific Super Resolution fMRI (STSS-SRfMRI) schemes. The STSS-SRfMRI scheme yielded a significantly smaller Dice coefficient than the raw fMRI (p = 0.00466). The median (interquartile range (IQR)) of raw fMRI and STSS-SRfMRI were 0.590 (0.408–0.735) and 0.490 (0.361–0.589), respectively.

Figure 6 illustrates the activated regions corresponding to the finger-tapping tasks. The activated regions obtained using the STSS-SRfMRI scheme had less overlap compared to those obtained using the raw unpreprocessed data. Figure 7 shows the Dice coefficients for the extracted thumb- and little finger-tapping related regions. The Dice coefficients were significantly smaller for the STSS-SRfMRI scheme (p = 0.00466).

## Discussion

In this study, we proposed a novel method based on a SRGAN that uses static T2*WI and subject-specific learning to improve functional resolution for fMRI. On visual assessment, the contrast of the activation map produced by the STSS-SRfMRI scheme was enhanced (Fig. 1). Quantitatively speaking, significantly fewer pixels were contained in the activation-related region derived from the STSS-SRfMRI processed data in comparison to the number obtained from the raw unpreprocessed data (Fig. 5). In addition, the Dice coefficients calculated for the activated regions corresponding to the two finger-tapping tasks were significantly lower for the STSS-SRfMRI processed data (Fig. 7). These results suggest that the STSS-SRfMRI method can improve functional resolution.

The thumb and little-finger related activation areas were narrower and more distinct in STSS-SRfMRI produced maps (Figs. 5, 6). This was quantitatively supported by the Dice coefficient analysis, where the values were significantly lower for the STSS-SRfMRI scheme in comparison to those obtained for the raw fMRI results (Fig. 7). These results suggest that the STSS-SRfMRI scheme may help to distinguish thumb and little-finger related activations more distinctly compared to the raw fMRI results. Previous studies have investigated finger somatotopy at both 3T^26,27^ and 7T^28^. While there was no gold-standard reference to verify the results at either field, it is likely that the 7T results will be more accurate because it is possible to image at higher resolution, which decreases the partial volume effect. Processing 3T fMRI data with the STSS-SRfMRI scheme might enable discrimination of activated areas that is comparable to that obtained using a 7T MRI scanner.

As noted above, the Dice coefficient tended to be lower for the STSS-SRfMRI results. However, there were seven individual cases where the Dice coefficient was found to be larger for the STSS-SRfMRI result. Closer examination of these cases found that the Dice coefficient was larger for the following reasons: (i) For two subjects, there was some misregistration of the motor cortex with the reference image, leading to some high CC pixels in the motor cortex being incorrectly discarded. It was not clear why the misregistration occurred, but after expanding the motor area using a region growing method^29,30^ the Dice coefficients were recalculated and found to be lower than the corresponding raw fMRI results. (ii) For one subject, although there were pixels within the brain with CCs over 0.5, the maximum CC in the motor area was less than 0.5. It is likely that in this case the subject did not adequately perform the tapping task. (iii) The activation area for one other subject was very broad, which suggests that accessory physical motion beyond the required task occurred. (iv) For three subjects, there were some artifacts in the T2*WI training images, which suggests that the corresponding SRGAN trained with those images was affected, and hence the generated STSS-SRfMRI images were defective.

Several studies have shown that using a SRGAN can improve the quality of medical imaging, and in particular MRI^11–13^. However, despite the improved appearance, few studies have suggested that MRI images reconstructed using a GAN are clinically or neuroscientifically significant^31^. An important feature of the present study is that the modified SRGAN not only generated acceptable higher resolution images, but maintained the embedded functional information.

Even though spatial filtering is widely used as a preprocessing step in the analysis of fMRI data, it could be argued that the super resolution networks in the STSS-SRfMRI scheme are just removing the smoothing effect of the filtering. To test this possibility the STSS-SRfMRI scheme was also applied to the unsmoothed data of all 30 subjects included in the final analysis (see Supplementary Fig. 2). It was found that the Dice scores without smoothing were lower for the STSS-SRfMRI processed data than for the raw fMRI data (0.417 (0.320–0.575) and 0.355 (0.238–0.457); data presented as median (interquartile range)). Although the median Dice scores for both schemes were lower than when filtering was used, a similar trend was found with the results of STSS-SRfMRI being significantly smaller than the raw fMRI results (p = 0.00000276).

One idea that could make the procedures proposed in this work more robust is to test the SRGAN trained for each subject on additional high-resolution T2*WI obtained from the same individual. Applying the STSS-SRfMRI scheme to the extra data would provide a first assessment of the accuracy of the results. Unfortunately, this idea was not applied in the present study because only one T2*WI data set was available for each subject.

One limitation of the present study is that there was no gold standard reference to verify the high-resolution functional maps generated using the proposed STSS-SRfMRI scheme. In the example shown in Supplementary Fig. 3, after analysis of the STSS-SRfMRI data the CC map for the thumb-tapping task appears to consist of several clusters of highly correlated pixels, whereas this feature was not observed for the raw fMRI maps. A previous study has determined that the activation regions in the primary motor cortex overlap for distinct movements of the fingers, wrist, and elbow^32^. Hence, it is possible that the clusters in Supplementary Fig. 3 reflect accessory movement during the thumb-tapping task. The absence of a gold standard reference prevented us from assessing whether this hypothesis was true or if it was simply an error due to the STSS-SRfMRI scheme generating incorrect EPI images.

Another possible limitation was that the T2*WI images obtained for subject-specific training were in 2D, which meant that a 2D GAN had to be used instead of a 3D GAN. As neural activity in the brain occurs in some 3D volume of tissue, a similar study using 3D images could increase the performance of STSS-SRfMRI in the future. Finally, as only healthy volunteers participated here, it was uncertain whether the proposed method is applicable for patients with neurological disorders. Clinical cases need to be studied in the future.

## Conclusions

In conclusion, we proposed a novel application of SR for fMRI using static T2*WI for training and applying subject-specific learning. The results suggest that the STSS-SRfMRI scheme has the potential to enhance the functional resolution of 3T fMRI by adequately increasing the spatial resolution of the original fMRI images.

## Supplementary Information

Supplementary Figures.

## Data Availability

The data supporting the findings of the current study are available from the corresponding author on reasonable request.

## References

[1] Pandarinathan G, Mishra S, Nedumaran AM, Padmanabhan P, Gulyás B (2018). The potential of cognitive neuroimaging: A way forward to the mind-machine interface. J. Imaging.

[2] Cheng K, Waggoner RA, Tanaka K (2001). Human ocular dominance columns as revealed by high-field functional magnetic resonance imaging. Neuron.

[3] Prasad A, Chaichi A, Kelley DP, Francis J, Ranjan Gartia M (2019). Current and future functional imaging techniques for post-traumatic stress disorder. RSC Adv..

[4] Bahrami K, Shi F, Rekik I, Gao Y, Shen D (2017). 7T-guided super-resolution of 3T MRI. Med. Phys..

[5] Goense J, Bohraus Y, Logothetis NK (2016). fMRI at high spatial resolution implications for BOLD-models. Front. Comput. Neurosci..

[6] Greenspan H (2009). Super-resolution in medical imaging. Comput. J..

[7] Kornprobst, P. *et al.* A superresolution framework for fMRI sequences and its impact on resulting activation maps. In *International Conference on Medical Image Computing and Computer-Assisted Intervention* 117–125 (Springer, 2003).

[8] Yang W (2019). Deep learning for single image super-resolution: A brief review. IEEE Trans. Multimed..

[9] Goodfellow, I. J. *et al.* Generative adversarial nets. In *Advances in Neural Information Processing Systems* 27 (2014).

[10] Ledig, C. *et al.* Photo-realistic single image super-resolution using a generative adversarial network. In *Proc. IEEE Conference on Computer Vision and Pattern Recognition* 4681–4690 (2017).

[11] Bing X, Zhang W, Zheng L, Zhang Y (2019). Medical image super resolution using improved generative adversarial networks. IEEE Access.

[12] Sood, R., Topiwala, B., Choutagunta, K., Sood, R. & Rusu, M. An application of generative adversarial networks for super resolution medical imaging. In *17th IEEE International Conference on Machine Learning and Applications, ICMLA 2018* 326–331 (IEEE, 2019).

[13] Jiang, X., Xu, Y., Wei, P. & Zhou, Z. CT image super resolution based on improved SRGAN. In *2020 5th International Conference on Computer and Communication Systems, ICCCS 2020* 363–367 (IEEE, 2020).

[14] Chavhan GB, Babyn PS, Thomas B, Shroff MM, Mark Haacke E (2009). Principles, techniques, and applications of T2*-based MR imaging and its special applications. Radiographics.

[15] Bandettini PA, Jesmanowicz A, Wong EC, Hyde JS (1993). Processing strategies for time-course data sets in functional mri of the human brain. Magn. Reson. Med..

[16] Rolls ET, Huang CC, Lin CP, Feng J, Joliot M (2020). Automated anatomical labelling atlas 3. Neuroimage.

[17] *SPM—Statistical Parametric Mapping*. https://www.fil.ion.ucl.ac.uk/spm/ (Accessed 2 November 2020)

[18] Ye W, Entezari A (2012). A geometric construction of multivariate sinc functions. IEEE Trans. IMAGE Process..

[19] Acharya, A. & Meher, S. Region adaptive unsharp masking based lanczos-3 interpolation for video intra frame up-sampling. In *2012 International Conference on Sensing TechSixthnology (ICST)* 57–62 (IEEE, 2012).

[20] Wang, X. *et al.* ESRGAN: Enhanced super-resolution generative adversarial networks. In *Proc. European Conference on Computer Vision (ECCV) Workshops* (Springer, 2018).

[21] Sorensen AT (1948). A method of establishing groups of equal amplitude in plant sociology based on similarity of species content and its application to analyses of the vegetation on Danish commons. Biol. Skar..

[22] Dice LR (1945). Measures of the amount of ecologic association between species. Ecology.

[23] Penfield W, Boldrey E (1937). Somatic motor and sensory representation in the cerebral cortex of man as studied by electrical stimulation. Brain.

[24] Jackson JH (1873). On the anatomical & physiological localisation of movements in the brain. Lancet.

[25] Kanda Y (2013). Investigation of the freely available easy-to-use software ‘EZR’ for medical statistics. Bone Marrow Transplant..

[26] Nelson AJ, Chen R (2008). Digit somatotopy within cortical areas of the postcentral gyrus in humans. Cereb. Cortex.

[27] Beisteiner R (2001). Finger somatotopy in human motor cortex. Neuroimage.

[28] Martuzzi R, van der Zwaag W, Farthouat J, Gruetter R, Blanke O (2014). Human finger somatotopy in areas 3b, 1, and 2: A 7T fMRI study using a natural stimulus. Hum. Brain Mapp..

[29] Haralick RM, Shapiro LG, Landesbibliothek TU (1992). Computer and Robot Vision.

[30] Gonzalez RC, Woods RE, Eddins SL (2009). Digital Image Processing Using MATLAB.

[31] Davatzikos C (2019). Machine learning in neuroimaging: Progress and challenges. Neuroimage.

[32] Donoghue J (2000). Plasticity and primary motor cortex. Artic. Annu. Rev. Neurosci..

