# Supplementary material for: Super-resolution generative adversarial networks with static T2*WI-based subject-specific learning to improve spatial difference sensitivity in fMRI activation
Source: Sci Rep. 2022 Jun 20;12:10319. doi: 10.1038/s41598-022-14421-5 (PMC9209532; doi:10.1038/s41598-022-14421-5)

**Supplementary Figure 1**

Experimental procedure for the finger tapping task. The cycle was repeated three times for the thumb and four times for the little finger. One of the cycles for the little finger was excluded for the analysis.


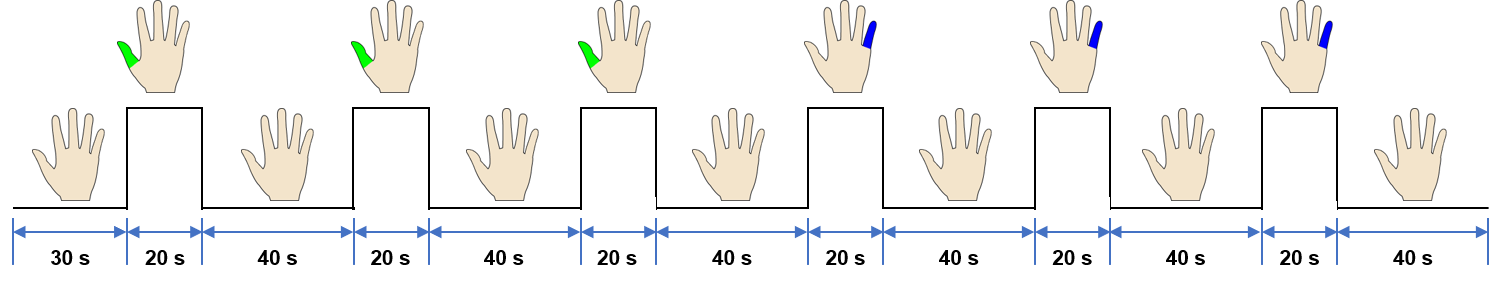


**Supplementary Figure 2**

Results of applying the raw fMRI and the Static T2*WI-based Subject-Specific Super Resolution fMRI (STSS-SRfMRI) procedures to the data without filtering. (a) Example of the axial activation maps obtained from the unfiltered data. To showcase the details, the area having the highest CC values in the primary motor cortex are magnified in the insets below. (b) Comparison of the Dice coefficients for the raw fMRI and the Static T2*WI-based Subject-Specific Super Resolution fMRI (STSS-SRfMRI) schemes. The STSS-SRfMRI scheme produced a significantly smaller Dice coefficient than the raw fMRI (p=0.00000276). The median (interquartile range (IQR)) of raw fMRI and STSS-SRfMRI were 0.417 (0.320–0.575) and 0.355 (0.238–0.457), respectively.


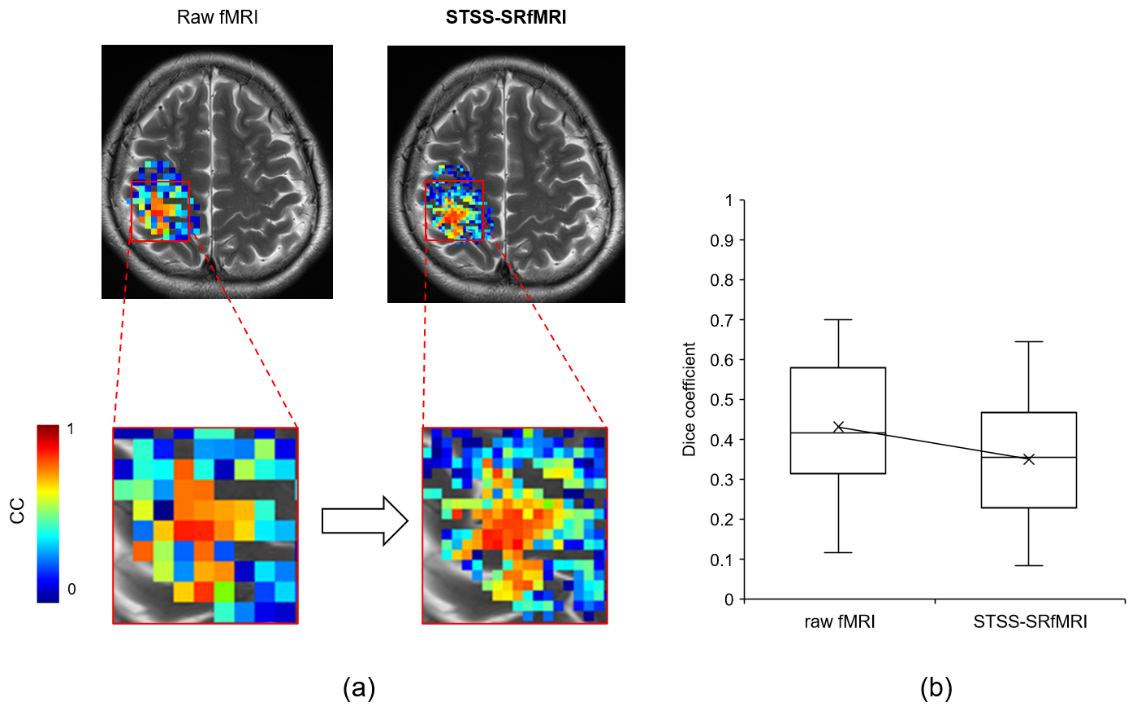


**Supplementary Figure 3**

Example of a cross-correlation (CC) map for the thumb-tapping activation task. The map calculated for the raw functional MRI (fMRI) data (left) indicates a broad connected pattern, that has the highest value in a concentrated core with lower values in the periphery. In contrast, the map calculated from the Static T2*WI-based Subject-Specific Super Resolution fMRI (STSS-SRfMRI) data (right) appears to consist of several almost disconnected clusters of highly correlated pixels.


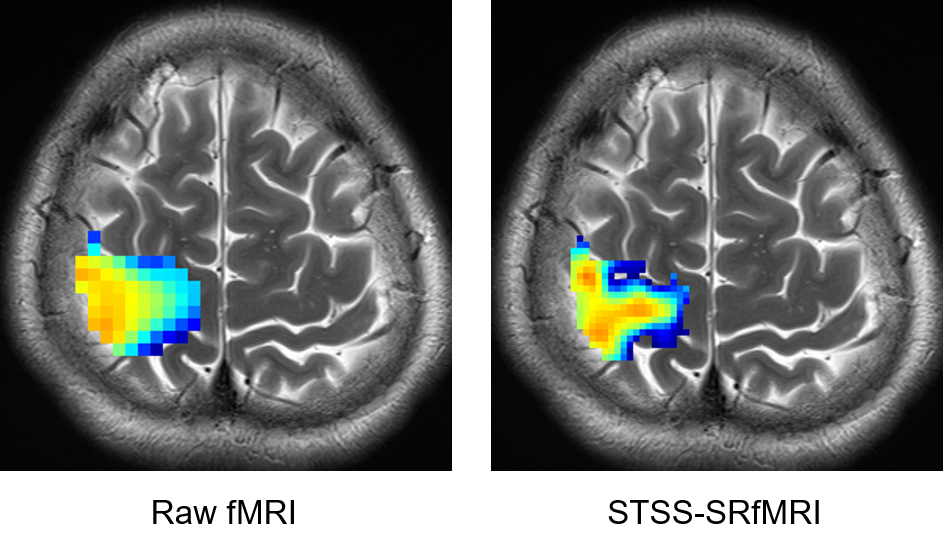

Supplement: Supplementary file 1 — Supplementary Figures. [file 41598_2022_14421_MOESM1_ESM.docx]
